# Adverse childhood experiences and psychopathology in adolescents from northern Chile: the moderating role of the attachment style

**DOI:** 10.1186/s41155-023-00273-z

**Published:** 2023-11-28

**Authors:** Cristián Pinto-Cortez, Gabriel Peñaloza-Díaz, Nicole Martínez, Sussan Díaz, Nicolle Valdovino, Margariett Zavala, Paola Muzatto-Negrón, Pamela Zapata-Sepúlveda

**Affiliations:** 1 Universidad de Tarapacá, Arica, Chile; 2 Programa de Doctorado en Psicología, Universidad de Tarapacá, Universidad Católica del Norte, Arica, Chile

**Keywords:** Adverse childhood experiences, Attachment style, Adolescence, Internalizing symptoms, Externalizing symptoms, Chile

## Abstract

**Background:**

Identifying the underlying mechanisms through which adverse childhood experiences affect (ACEs) the mental health of adolescents is of paramount importance for disease prevention in later stages of life.

**Objective:**

The present study examines the relationship between ACEs and psychopathology in adolescents from northern Chile and how attachment style (abandonment anxiety and intimacy avoidance) may moderate this relationship. A total of 154 schooled adolescents aged 12 to 17 (*M* = 15.08, *SD* = 1.64) completed a series of self-report questionnaires including the Adverse Childhood Experience Questionnaire (ACEs), Experiences in Close Relationships- Relationship Structures (ECR-RS), and Youth Self Report (YSR-18).

**Results:**

The data analysis was carried out using SPSS version 25, which included descriptive analysis, one-way ANOVA, and Spearman correlation analysis. To address moderation analysis, the PROCESS macro extension version 4.1 was employed. In this process, the bootstrap method was applied to construct confidence intervals, and the pick-a-point approach was used to define the levels of the moderating variable. According to the results, 80.3% of the sample experienced one or more ACEs, and 16.4% reported experiencing at least three. Furthermore, the variables under study exhibited significant correlations with each other, except for intimacy avoidance, which showed no correlation with ACEs (*rho* = -0.10; *p* = 0.273). When considering abandonment anxiety as a moderating variable, the direct effect of ACEs on externalizing symptoms showed statistically significant changes (*β* = 0.60, *p* = 0.03). No other moderating effects were found according to the proposed models.

**Conclusion:**

In childhood, the accumulation of ACEs is associated with the development of psychopathology in adolescents from northern Chile, specifically with the presence of internalizing and externalizing symptoms. These findings suggest that lower levels of abandonment anxiety could mitigate the effects of ACEs on adolescent psychopathology, while higher levels of abandonment anxiety could exacerbate these effects on psychopathology.

## Introduction

Adverse childhood experiences (ACEs) are defined as potentially traumatic events that can have harmful and long-lasting effects on the health and well-being of individuals (Antoniou et al., 2023; Boullier & Blair, 2018; González-Araya et al., 2023; Vyas et al., 2023). Various studies on this topic have been developed in different countries, in which the close relationship between ACEs and the adverse effects on the physical and mental health of the population has been proven (Fernández et al., 2019; Graf et al., 2021; Hughes et al., 2017; Petruccelli et al., 2019; Schroeder et al., 2021). Some ACEs that have been addressed in previous studies are child maltreatment, caregiver drug use, sexual abuse, and caregiver mental health problems, which have shown robust evidence with a wide range of physical issues such as overweight, diabetes, digestive diseases, respiratory diseases, smoking, and teenage pregnancy and also with mental health problems such as anxiety, depression, drug or alcohol use, revictimization, perpetration of violence, suicide attempts (Hughes et al., 2017) and hallucinations (Petruccelli et al., 2019), among other psychological conditions. In the specific case of the relationship between ACEs and psychopathology, research conducted with adolescents in Finland revealed that ACEs were associated with suicidality and psychiatric disorders in this population. Specifically, the study detected that adolescents hospitalized for psychiatric reasons reported a higher number of ACEs compared to adolescents in nonclinical samples. More specifically, while 20% of the hospitalized adolescents had been exposed to at least four ACEs in the community samples, this proportion was only 2%. The most frequent ACEs reported by adolescents in the study were caregiver divorce and caregiver mental health problems. Furthermore, sexual abuse was the most significant event associated with psychiatric psychopathology (Rytilä-Manninen, 2018). Other studies, also with adolescents (Anderson et al., 2022; Henry et al., 2021; Liu et al., 2023), found a direct relationship between ACE and externalizing psychopathological symptoms such as anger, aggressive behavior, and breaking-rule behavior, oppositional defiant conduct, and impulsivity/hyperactivity.

In Latin America, some studies have been conducted on this topic, especially on the prevalence of ACE in adolescents and its link with emotional and neurophysiological functioning. Gonçalvez et al. (2016) assessed the prevalence of ACEs in 3.951 adolescents from Brazil. Seven types of ACEs were assessed in those adolescents up to 18 years old: physical abuse, sexual abuse, physical neglect, emotional neglect, domestic violence, parental separation, and parental death. The most common ACE was parental separation (42%), followed by emotional neglect (19.7%) and domestic violence (10.3%). Approximately, 85% of the adolescents experienced at least one ACE, and females reported more adversities. Several socioeconomic, demographic, and family-related characteristics were associated with the occurrence of ACEs, e.g., non-white skin color, low family income, low maternal schooling, the absence of mother’s partner, maternal smoking, and poor maternal mental health. Moreover, León (2019) found that ACEs are frequent events in Colombian adolescents (99.2% have suffered at least one ACE in their lifetime) and are particularly higher in females than males. In addition, the study showed that the accumulation of adverse experiences such as abuse, family violence, and school problems increased the risk of presenting mental health problems such as the externalizing symptoms and anxiety.

In Chile, a study of children and adolescents who experienced different types of maltreatment (sexual, physical, psychological, and neglect) showed a higher prevalence of psychopathology in the adolescent group, with the presence of mood disorders and disruptive disorders, as well as substance use disorders and schizophrenia (Riquelme et al., 2020). Additionally, the rates of violence-related ACEs experienced by children and adolescents in Chile are significantly elevated, where 92.6% of them reported at least one type of victimization in their lifetime (Pinto-Cortez et al., 2022; UNICEF, 2015).

### Attachment and adolescence

Attachment is a particular type of affective bond that aims to ensure the survival of human beings. The attachment was described by Bowlby (2003), who argued that infants have a biological predisposition to develop a motivational and behavioral system that promotes proximity to their caregivers, with the ultimate goal of obtaining protection in situations of threat, vulnerability, and stress. This affective bond, accompanied by a sensitive and consistent response from the caregivers, would be a determining factor for developing healthy interpersonal bonds maintained from childhood to adulthood. Nowadays, attachment theory has a high heuristic value in the field of developmental psychology since supported by empirical evidence; it has managed to position the attachment bond as the fundamental basis for the cognitive, affective, and social development of children and adolescents (Bernier et al., 2015; Bohlin et al., 2012; Cassidy, 1994; Groh et al., 2017; Sroufe, 2005). Thus, some authors have suggested that a secure attachment in childhood (Ainsworth et al., 1978) could constitute a protective factor against developing mental health problems or psychopathology in childhood and adulthood (Sund & Wichstrøm, 2002). Conversely, an insecure attachment could be a risk factor (although not the only cause) for developing mental health problems or psychopathology in adolescence or adulthood (Kerns & Brumariu, 2014; Leben Novak et al., 2023). In the same vein, it has been shown that secure attachment could have some influence in the prevention of multiple problematic issues such as violent behavior (Franke, 2000), post-traumatic stress (Ensink et al., 2021), behavioral problems in children (Barone et al., 2016; Edwards et al., 2006), dementia (Walsh et al., 2019), and even cellular aging (Dagan et al., 2018). Moreover, studies on insecure attachment have shown its influence as a risk factor in suicide (Miniati et al., 2017; Sheftall et al., 2014), anxiety (Kerns & Brumariu, 2014), technology addiction (Remondi et al., 2020), dating violence (Ponti & Tani, 2019), depression (Spruit et al., 2020), and other mental health problems (Ensink et al., 2020).

Adolescence is a period of particular interest for attachment studies, considering several neurobiological, psychological, and social transformations occur at this stage, including changes at the bonding level (Krauskopof, 1999). During adolescence, attachment styles acquire a fundamental role as adolescents expand their social circle, decrease emotional closeness with their caregivers, and experience new friendships and couple relationships (Oliva Delgado, 2011). Within this context, adolescents manifest a distancing from parents and a search for autonomy, which affects the expressions of affection, the amount of time spent with their caregivers, and the increase in their privacy (Oliva Delgado, 2011). Likewise, other significant attachment bonds appear, including peers or first romantic partners (Dykas et al., 2008). Notwithstanding, caregivers remain an essential source of emotional support in stressful situations (Portilla-Saavedra et al., 2022). In this context, the study of attachment in adolescence is relevant as it is a stage of change, mainly because it is a stage of low stability in close attachment bonds (Jones et al., 2018), channeled and stabilized as they reach adulthood. Adolescence is a risky stage but also full of opportunities as new affective bonds appear that could be potentially protective.

**Fig. 1:**
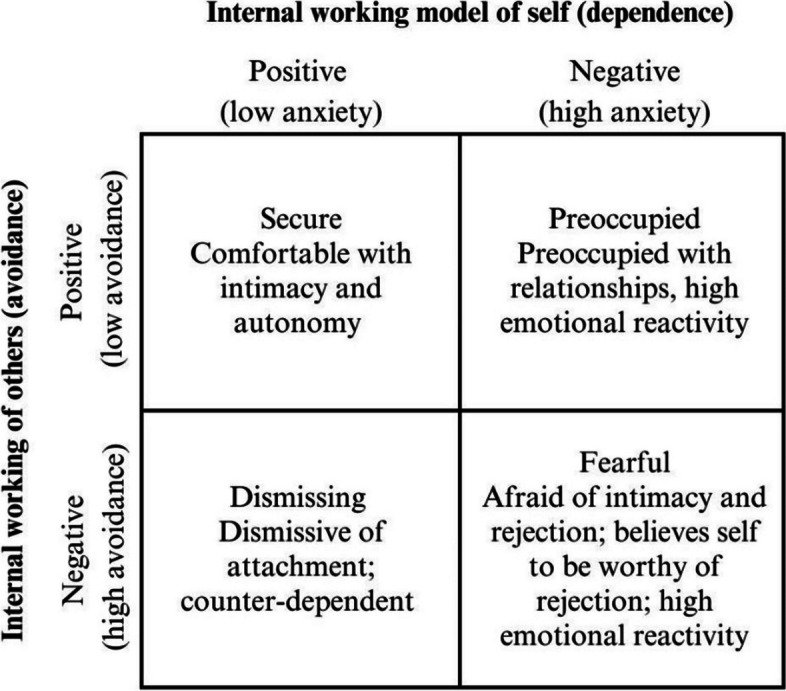
Bartholomew’s four-category model of attachment (Bartholomew & Horowitz, 1991) based on Ma (2006)

To understand the characterization of adolescent attachment in a better way, Bartholomew’s model (1990) will be used in this study, which includes a dimensional and prototypical classification with characteristics associated with each type of attachment, highlighting the characteristics of adolescents with high/low anxiety and high/low avoidance associated with each attachment styles (Bartholomew & Horowitz, 1991). In Fig. 1, the model is described.

According to Camps-Pons et al. (2014), in addition to the categorical and dimensional classification of attachment, it is also helpful to consider the greater or lesser presence of anxiety and avoidance in the different attachment types. Secure and avoidant attachment types (positive view of the self) experience less anxiety in interpersonal relationships and more trust, whereas anxiety, fear of rejection, is more significant in preoccupied and fearful attachments (negative view of the self). Concerning avoidance, in a secure and preoccupied attachment (positive outlook of others), there would be less interpersonal distance, and proximity would be sought more. In contrast, in avoidant and fearful styles (negative view of others), relationships with greater distance and less intimate would be preferred.

### Adverse experiences, attachment, and psychopathology

Various studies in different countries have found an influence of attachment between ACEs and psychological well-being/discomfort (Corcoran & McNulty, 2018; Howard et al., 2023; Snyder et al., 2023). For example, Corcoran and McNulty (2018) found that ACEs were strongly associated with psychological distress/well-being, and that, at the same time, these associations were mediated by attachment. Specifically, the higher the anxious attachment, the higher the psychological distress and the lower the subjective well-being. Another study by Derin et al. (2022) reported that childhood emotional neglect and income level (considered ACEs) predicted a higher risk for anxiety disorder in adolescence. In this research, however, attachment styles did not significantly contribute to predicting adolescent anxiety disorders. Similarly, Yang and Perkins (2021), when studying the mediating role of attachment between childhood adversity and criminal thinking, found that the higher the adversity in childhood, the higher the presence of criminal thinking. Nevertheless, anxiety in attachment was not associated with criminal thinking, nor did it mediate the influence between childhood adversity and criminal thinking.

Conversely, Wilke et al. (2020) found that ACEs in childhood were related to greater problematic use of technologies/media, and that both anxious attachment and avoidant attachment independently mediated the relationship between childhood adverse experiences and problematic use of media/information technologies. Specifically, the avoidant attachment was associated with less problematic use of media/information technologies and anxious attachment with more problematic use of technology.

Alternatively, in the field of psychopathology, a study conducted by Elling et al. (2022) in Germany aimed to evaluate attachment styles, notably the fearful attachment style (high anxiety and high avoidance) and its relationship with social anxiety disorder and major depression. The study, conducted with a clinical sample of 472 adult participants, concluded that fearful attachment mediated the relationship between childhood adversities, social anxiety disorders, and major depression by aggravating the intensity of these pathologies (Elling et al., 2022).

Nonetheless, despite the findings obtained in these studies, there are some limitations. For example, it has been pointed out that in some of these investigations, only one attachment style is evaluated and, to a lesser extent, the other attachment styles (Elling et al., 2022). Also, the mental health indicators used have been diverse and occasionally are oriented to evaluate psychopathology specifically (Corcoran & McNulty, 2018).

In Chile, a study related attachment styles with psychopathology in a community sample (*N* = 1.042) of adolescents from northern Chile. The results showed that most adolescents presented avoidant attachment to caregivers (30.2%), followed by preoccupied attachment (28.4%), fearful attachment (22.9%), and, lastly, secure attachment (18.5%). In addition, fearful attachment was related to all psychopathological symptoms considered in the study (Pinto-Cortez et al., 2018). However, this study did not consider ACE as a study variable, nor did it consider psychopathology from a dimensional model. To be specific, there are two distinct and substantial categories of psychological problems identified through empirical studies (Achenbach et al., 1987): (a) internalizing, which represents anxious symptoms, depressive, and somatic problems, and (b) externalizing, which includes problems related to impulsivity, disruptive behaviors, and substance abuse (Achenbach et al., 2016). However, despite the importance of these studies, in Chile, no studies relate ACE with attachment and psychopathology and consider psychopathology from a dimensional perspective.

### The current study

From the literature reviewed, most studies on ACEs have had difficulties in operationalizing the concept, considering it as a summative construct where each adverse experience would have the same weighting/effect on the eventual consequences on the development of individuals (Cross et al., 2023; Finkelhor, 2018). Without considering, for example, the difference between adverse experiences may be more invasive, such as sexual abuse, or others less invasive, such as a parent’s mental illness or incarceration. Additionally, fewer studies have incorporated attachment as a third variable to assess its moderating/mediating effect between ACEs and mental health problems (Corcoran & McNulty, 2018). Only a few studies on this topic have incorporated all attachment types (e.g., anxious and avoidant attachment together) (Elling et al., 2022).

As discussed above, another gap is related to the mental health indicators used, as these have been diverse. For example, some use general mental health criteria, and other research uses broad criteria on well-being or other indicators. Furthermore, most of these studies have been carried out with retrospective samples of adults, with the limitations inherent to this type of work (bias due to memory effect) and the underrepresentation of children and adolescents from different cultural contexts.

In this context, the present study aimed to resolve some of these issues, incorporating a concrete and differentiating conceptualization of adverse experiences, leaving out those that the literature has pointed out as the most serious, such as sexual abuse and physical mistreatment, omitting a simple summative effect, considering a concrete indicator of psychopathology, and also incorporating the assessment of attachment in dimensional terms (spectrum of security/insecurity and anxiety/avoidance). Likewise, the study was conducted with adolescents in a context different from those in which attachment has traditionally been studied in this age group.

Thus, the present research is academically relevant since it contributes theoretically to the approach of ACEs, attachment theory, and psychopathology studies in adolescence. At the social level, the results derived from this study can generate actions for the prevention of adverse experiences in childhood and, consequently, contribute to mental health problems specific prevention in adolescents.

In this context, the main objective of this study was to assess the moderating effect of attachment between ACEs and psychopathology in adolescents from northern Chile. Two main questions were outlined as follows:

1. What is the relationship between adverse childhood experiences and psychopathology?
2. How does attachment insecurity contribute to the relationship between adverse childhood experiences and psychopathology?

Based on the literature reviewed, it is hypothesized as follows:

- H1: Adverse childhood experiences increase the level of psychopathology in adolescents.
- H2: The negative impact of adverse childhood experiences on psychopathology (internalizing symptoms) will be higher and the more substantial the attachment insecurity (abandonment anxiety) reported by adolescents.
- H3: The negative impact of adverse childhood experiences on psychopathology (externalizing symptoms) will be higher and the stronger the attachment insecurity (abandonment anxiety) adolescents report.
- H4: The negative impact of adverse childhood experiences on psychopathology (internalizing symptoms) will increase and the deeper the attachment insecurity (intimacy avoidance) adolescents report.
- H5: The negative impact of adverse childhood experiences on psychopathology (externalizing symptoms) will increase and the deeper the attachment (intimacy avoidance) adolescents report.

## Method

### Participants

The initial sample consisted of 154 schooled adolescents from three schools in the city of Arica, in the extreme north of Chile. It is important to note that a convenience sampling method was utilized for participant recruitment. Of these, 82 were females, and 72 were males, ages 12 to 17 (*M* = 15.08, *SD* = 1.64). The students belonged to public, private, and subsidized schools. To be included in the study, adolescents had to be under 18 and enrolled in the corresponding courses at the selected schools. Participants who did not provide their own and caregivers’ informed consent were excluded from the study. All the schools were coeducational (without gender restrictions), with an average of 773 annual students, and were located in urban areas of the city. Additionally, the schools admitted a wide variety of students from different socioeconomic backgrounds, ethnicities, nationalities, and cultures.

### Measures

#### Sociodemographic

A questionnaire was designed that included various sociodemographic elements and biographical data of the participants, such as age, sex, level of studies, and educational center.

#### ACEs

A modified version of the Adverse Childhood Experience Questionnaire for Adults in its Spanish version was utilized (California Department of Health Care Services, 2023). The ACEs Questionnaire is a publicly accessible self-administered questionnaire developed by the Clinical Advisory Committee of the Surgeon General of California. Its purpose is to assess the presence or absence of 10 types of adverse experiences lived before the age of 18. The questionnaire is scored on a scale from 0 to 1, where 0 indicates the absence and 1 the presence of the experienced event. Higher scores indicate a more significant number of adverse experiences before age 18. The questions are designed to identify various experiences such as parental divorce, mental illnesses in caregivers, exposure to family violence, parental neglect, imprisonment of a household member, and cohabitation with individuals with alcohol- or drug-related issues. However, due to the age range of the participants and the study’s focus on a community sample of high school students, the decision was made to exclude certain items from the original questionnaire. This was done to prevent generating unnecessary distress or discomfort in adolescents. Therefore, questions related to cohabitation with a suicidal person, experiencing unwanted sexual contact, direct physical violence, and verbal violence, were excluded. The final questionnaire consisted of 6 items. An accumulated ACE score (range = 0–6) was calculated by summing the 6 dichotomized ACE indicators. Consistent with previous studies (Flaherty et al., 2013; Haatainen et al., 2003; Von Cheong et al., 2017), all adolescents were classified into four groups based on their accumulated ACE score, i.e., 0, 1, 2, and ≥ 3 ACE.

#### Experiences in close relationship-relationship structures (ECR-RS) (Fraley et al., 2011)

An adapted Spanish version of the experiences in close relationships-relationship structures (ERC-RS) instrument, validated in a Chilean population (Tay Karapas et al., 2016), was used in this study. This scale consists of nine items distributed across two subscales, avoidance, and anxiety, with items 1 to 6 assessing the dimension of intimacy avoidance and items 7 to 9 evaluating the dimension of abandonment anxiety. The same nine items were employed to measure attachment in four relational domains: towards the mother, father, romantic partner, and best friend, yielding independent scores for each domain in the two described dimensions. Participants responded using a 7-point Likert scale ranging from 1 (totally disagree) to 7 (totally agree). Higher scores on this instrument are interpreted as higher levels of anxiety and avoidance, thus indicating greater attachment insecurity. The instrument demonstrated satisfactory internal consistency indices, as assessed by the Cronbach’s alpha coefficient for each dimension (anxiety = 0.90; avoidance = 0.76).

For the purposes of this study, both scales assessing the dimensions of attachment towards the mother and father were utilized, resulting in a total of 18 items. Additionally, the attachment dimensions for the mother and father were averaged to generate an overall score for abandonment anxiety and intimacy avoidance.

#### Youth Self-Report (YSR-18) (Achenbach, 1991)

In this study, the Chilean version of the Youth Self-Report (YSR/11–18; Leiva Bahamondes & Rojas Andrade, 2018) was employed. This instrument consists of two parts. The first part includes seven semi-structured and three open-ended questions that measure social skills or competencies. The second part comprises 112 multiple-choice items, of which 16 explore the frequency of adaptive or prosocial behaviors, while the rest assess a wide range of problematic behaviors. All items in this second section are responded to by the subject based on their applicability and frequency, selecting 0 when “not true,” 1 when "true in some way, sometimes" and 2 when “very true or true very often.”

For the purposes of this study, the section related to emotional and behavioral problems, composed of 112 items, was utilized. This instrument adheres to the assumption of continuity of abnormal experiences and behaviors, allowing each case to be placed within a set of psychopathological dimensions (Lemos Giráldez et al., 2002). It consists of eight primary scales or first-order syndromes (depression/anxiety, aggressive behavior, delinquent behavior, thought problems, somatic complaints, social problems, attention problems, and social withdrawal), two secondary scales or second-order syndromes (internalizing, externalizing), and one tertiary or overall scale.

### Procedure

Following the approval of the research protocol by the University Scientific Ethical Committee, coordination with the schools and contact with the selected students and their caregivers was carried out. The schedules and places where the application would take place were planned. Each student and their caregivers had to sign a letter of informed consent to participate in the research. In the second stage, the assessment instruments were applied by senior psychology students trained for this study. Specifically, the instruments were applied online and in the classrooms during lesson hours, agreed with the school center, and without the teacher’s presence. The participants followed the instructions, and refusal responses to the instruments were not obtained. The confidentiality of the answers and, likewise, of the data confidentiality were informed to the participants. The survey duration was between 30 and 50 min, and the collection data was collected in 4 months. The research protocol included telephone and e-mail contact numbers in case a student needed to talk to a professional about the consulted experiences or if they needed mental health support. Only one student requested mental health support, and she and her mother were referred to specialized mental health services.

### Data analysis

Initially, exclusion criteria were applied to the total sample of participants, considering the percentage of incomplete responses in the questionnaire. Those cases with a percentage of missing values exceeding 20% were decided to be eliminated to ensure the integrity and validity of the collected data. Additionally, participants identified as cases with atypical responses were excluded from the final sample. That is, cases were identified where there was a lack of variation in their response choices, as repetitive patterns or consistent responses throughout the questionnaire were detected. These cases were considered atypical due to the limited variability in their responses, which could indicate a lack of attention or systematic bias in their answers. To identify these cases, the standard deviation was calculated for each participant in the questionnaire. Those with a very low or zero standard deviation were excluded from the sample, as it indicated invariable responses. As a result, the final sample consisted of 122 adolescents. The detailed characteristics of the participants are shown in Table 1.

**Table 1:** Comparison of sociodemographic characteristics, abandonment anxiety, intimacy avoidance, internalizing symptoms, and externalizing symptoms according to the cumulative number of ACEs in adolescents

Characteristics	Number of ACEs (*n* = 122)				
	0 (*n* = 24)	1 (*n* = 52)	2 (*n* = 26)	≥ 3 (*n* = 20)	*p*-value for group difference
Sex, *n* (%)					0.160
Boys	15 (62.5%)	28 (53.8%)	12 (46.2%)	6 (30.0%)	
Girls	9 (37.5%)	24 (46.2%)	14 (53.8%)	14 (70.0%)	
Age (years), mean ± SD	15.17 ± 1.6	14.94 ± 1.7	15.31 ± 1.6	14.85 ± 1.6	0.736
Type of establishment, *n* (%)					0.751
Public	16 (66.7%)	27 (52.9%)	13 (50.0%)	12 (60.0%)	
Private	7 (29.2%)	20 (38.5%)	9 (34.6%)	7 (35.0%)	
Subsidized	1 (4.2%)	5 (9.6%)	4 (15.4%)	1 (5.0%)	
Grade^a^					0.780
6° básico (sixth grade)	2 (8.3%)	4 (7.7%)	1 (3.8%)	2 (10.0%)	
7° básico (seventh grade)	1 (4.2%)	8 (15.4%)	4 (15.4%)	2 (10.0%)	
8° básico (eighth grade)	5 (20.8%)	7 (13.5%)	4 (15.4%)	2 (10.0%)	
1° medio (ninth grade)	1 (4.2%)	5 (9.6%)	5 (19.2%)	5 (25.0%)	
2° medio (tenth grade)	4 (16.7%)	6 (11.5%)	1 (3.8%)	3 (15.0%)	
3° medio (eleventh grade)	6 (25.0%)	15 (28.8%)	5 (19.2%)	4 (20.0%)	
4° medio (twelfth grade)	5 (20.8%)	7 (13.5%)	6 (23.1%)	2 (10.0%)	
Abandonment anxiety, mean ± SD	3.06 ± 1.8	3.70 ± 1.7	4.57 ± 1.4	4.81 ± 1.5	.001
Intimacy avoidance, mean ± SD	3.37 ± 1.5	3.85 ± 1.3	3.62 ± .8	3.23 ± .8	0.152
Internalizing symptoms, mean ± SD	16.33 ± 10.0	20.42 ± 9.8	28.35 ± 11.1	29.30 ± 9.9	< .001
Externalizing symptoms, mean ± SD	5.75 ± 4.1	9.10 ± 4.6	12.62 ± 6.1	15.70 ± 8.5	< .001

*ACEs* adverse childhood experiences, *SD* standard deviation. *p*-value for group difference was obtained using one-way ANOVA and *χ*^2^ test. ^a^In this s tudy, we included students from 6° básico (sixth grade) to 4° medio (twelfth grade), which corresponds to primary and secondary education in the Chilean educational system. In Chile, primary education typically consists of 8 years, starting from 1 to 8° básico, while secondary education spans 4 additional years from 1 to 4° medio

Descriptive statistics were reported as mean ± standard deviation (SD) for continuous data and frequency (percentage) for categorical data. One-way ANOVA compared characteristics across the four ACE groups for continuous variables and *χ*^2^ test for categorical variables.

The association between ACE, attachment dimensions, and psychopathological symptoms was evaluated through correlation analysis. For the purposes of this study and to conduct subsequent analyses, the sum of the 6 ACEs was used as a continuous variable. The nonparametric Spearman’s rho test was employed to measure the correlation coefficient between the variables, as they did not meet the assumptions of normality and homoscedasticity.

**Fig. 2:**
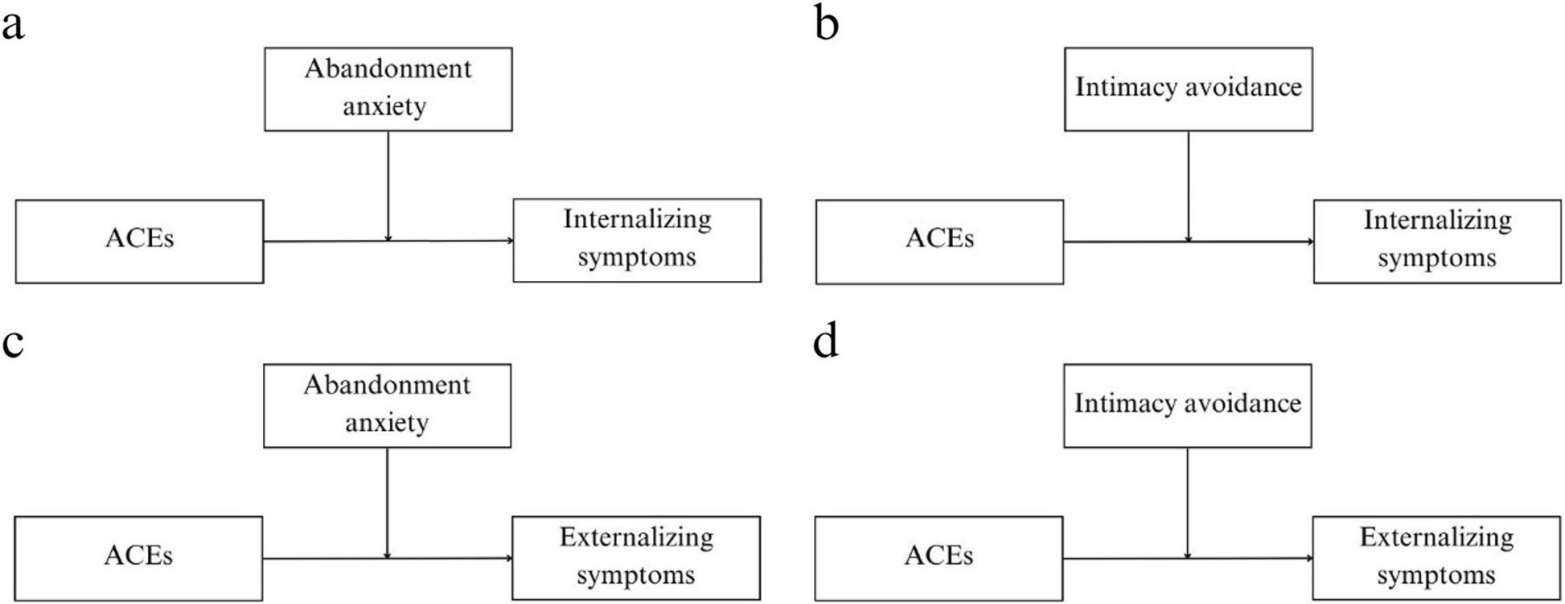
Moderation diagrams showing the effect between ACEs and internalizing symptoms moderated by **a** abandonment anxiety or **b** intimacy avoidance and the effect between ACEs and externalizing symptoms moderated by **c** abandonment anxiety or **d** intimacy avoidance

Subsequently, moderation analyses were performed to explore whether the relationship between ACEs (X) and internalizing (Y) or externalizing (Y) symptoms was moderated by abandonment anxiety (W) or intimacy avoidance (W) (Fig. 2). Analyses were conducted separately for both outcome and moderating variables. The PROCESS macro extension version 4.1 for IBM SPPS was used, and the procedures described by Hayes (2022) were followed. The aim was to determine whether attachment insecurity moderated the effect between ACEs and psychopathological symptoms (internalizing-externalizing) through the analysis of confidence intervals (without the inclusion of zero) and tests of statistical significance (*p* < 0.05). Given the distribution of the study variables, the bootstrap method with 10.000 iterations was used to construct confidence intervals for the estimated parameters (Hayes, 2022; MacKinnon, 2008). A conditional effect of insecurity on attachment was investigated using the pick-a-point approach (Bauer & Curran, 2005; Rogosa, 1980) to establish low (16th percentile), medium (50th percentile), and high (84th percentile) levels for both abandonment anxiety and intimacy avoidance.

## Results

### Descriptive statistics

As presented in Table 1, approximately 80.3% (*n* = 98) of the adolescents had experienced one or more ACEs, and 16.4% (*n* = 20) were exposed to at least three ACEs. Adolescents who had experienced three or more ACEs were younger and more likely to exhibit abandonment anxiety and internalizing and externalizing symptoms compared to adolescents without ACE exposure. Furthermore, as the cumulative number of ACEs increased, there was an observed rise in the mean scores of abandonment anxiety and psychopathological symptoms.

### Correlation analysis

Table 2 summarizes the results of the correlation Spearman analysis for all study variables. As expected, the vast majority of the variables were significantly correlated with each other. ACEs showed moderate and statistically significant correlations with abandonment anxiety (rho = 0.37, *p* < 0.001), internalizing symptoms (rho = 0.42, *p* < 0.001), and externalizing symptoms (rho = 0.49, *p* < 0.001). However, intimacy avoidance was not statistically correlated with ACEs (rho =  − 0.10, *p* = 0.273).

**Table 2:** Spearman’s rho bivariate correlations

	2	3	4	5
1. ACEs	0.37 (*p* < .001)	− 0.10 (*p* = 0.273)	0.44 (*p* < .001)	0.49 (*p* < .001)
2. Abandonment anxiety		− 0.15 (*p* = 0.098)	0.25 (*p* = .006)	0.29 (*p* = .001)
3. Intimacy avoidance			0.11 (*p* = 0.243)	− .01 (*p* = .911)
4. Internalizing symptoms				0.57 (*p* < .001)
5. Externalizing symptoms				

*ACEs* adverse childhood experiences

### Moderation analysis

**Fig. 3:**
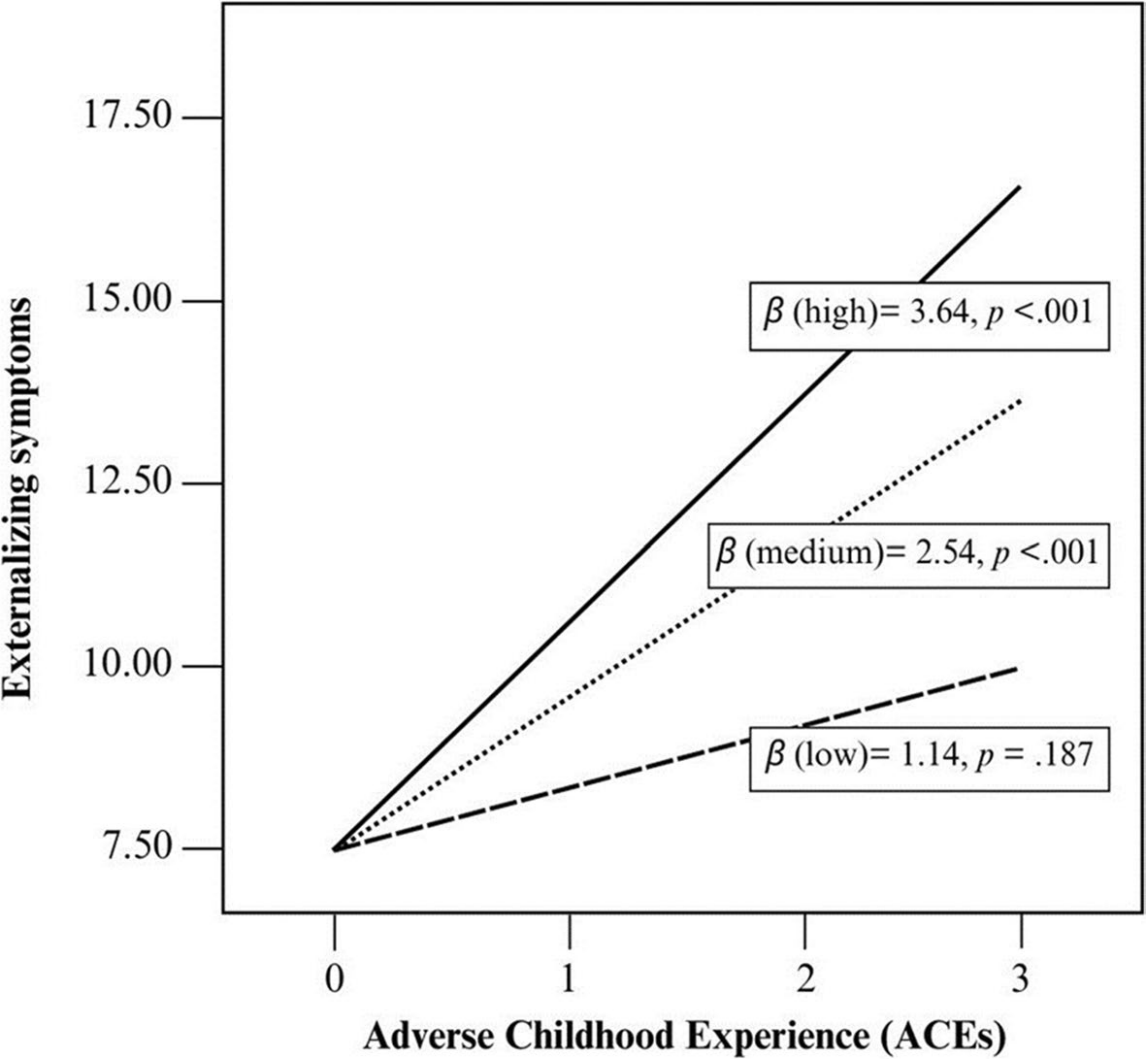
Graphic of the conditional effect of abandonment anxiety levels (low, medium, and high) on the relationship between ACEs and externalizing symptoms

Tables 3 and 4 present the results of the moderation analysis. When considering abandonment anxiety as the moderating variable, the direct effect of ACEs on externalizing symptoms showed statistically significant changes (*β* = 0.60, *p* = 0.03; *LLCI* = 0.05, *ULCI* = 1.15). Furthermore, it was found that the change in R-squared associated with the interaction effect was also significant (*R*^2^ chng = 0.028, *F* = 4.70, *p* = 0.032). Specifically, adolescents’ abandonment anxiety levels influenced the relationship between ACEs and externalizing symptoms. Figure 3 displays a graph of the conditional effect of abandonment anxiety levels (low, medium, and high) on the relationship between ACEs and externalizing symptoms. When abandonment anxiety was low (16th percentile = 1.67), the effect of ACEs on externalizing symptoms disappeared (*p* = 0.187). However, when abandonment anxiety was moderate (50th percentile = 4.0, *p* < 0.001) and/or high (84th percentile = 5.83, *p* < 0.001), the impact of ACEs on externalizing symptoms was greater and statistically significant. These findings suggest that attachment insecurity, represented by abandonment anxiety, acts as a moderator in the relationship between adverse experiences and externalizing symptoms in adolescents. No other moderation effects were found according to the proposed models.

**Table 3:** Moderation analysis: changes in the R-square for the interaction effect

Model	
*X* = ACEs *Y* = Internalization *W* = Abandonment anxiety	I: *R* = 0.44, *R*^2^ = 0.19, *F* (118) = 9.49, *p* > .001, *MSE* = 102.84 II: *R*^2^-chng = .00, *F* (118) = 0.18, *p* = 0.672
*X* = ACEs *Y* = Internalization *W* = Intimacy avoidance	I: *R* = 0.46, *R*^2^ = 0.22, *F* (118) = 10.81, *p* > .001, *MSE* = 100.13 II: *R*^2^-chng = .02, *F* (118) = 3.35, *p* = 0.070
*X* = ACEs *Y* = Externalization *W* = Abandonment anxiety	I: *R* = 0.56, *R*^2^ = 0.31, *F* (118) = 17.77, *p* > .001, *MSE* = 29.42 **II:** ***R***^**2**^ **-chng = .03,** ***F*** **(118) = 4.70,** ***p* = *.032***
*X* = ACEs *Y* = Externalization *W* = Intimacy avoidance	I: *R* = 0.51, *R*^2^ = 0.26, *F* (118) = 13.79, *p* > .001, *MSE* = 31.62 II: *R*^2^-chng = .00, *F* (118) = 4.75, *p* = 0.492

The statistically significant interactions were highlighted in bold. I, model summaries and II, higher-order tests of unconditional interactions. *MSE* mean-squared error, *R*^2^ R-square, *R*^2^-chng change in R-square, *p* p-value, *ACEs* adverse childhood experiences

**Table 4:** Linear models for predictors of psychopathological symptoms

Model		*β*	*SE*	*t*	*p*	*LLCI*	*ULCI*
*X* = ACEs *Y* = Internalization *W* = Abandonment anxiety	Constant	14.40	3.31	4.35	.00	7.85	20.96
	Adverse experiences	2.72	2.36	1.15	0.25	− 1.96	7.40
	Abandonment anxiety	0.82	0.84	0.97	0.33	− 0.85	2.49
	*X* × *W*	0.22	0.52	0.42	0.67	− 0.80	1.24
*X* = ACEs *Y* = Internalization *W* = Intimacy avoidance	Constant	6.20	4.25	1.46	0.15	− 2.22	14.62
	Adverse experiences	9.47	2.92	3.25	.00	3.70	15.25
	Intimacy avoidance	3.06	1.14	2.67	.01	0.79	5.32
	*X* × *W*	− 1.55	0.85	− 1.83	.07	− 3.23	0.13
*X* = ACEs *Y* = Externalization *W* = Abandonment anxiety	Constant	6.68	1.77	3.77	.00	3.17	10.19
	Adverse experiences	0.14	1.26	0.11	0.91	− 2.37	2.64
	Abandonment anxiety	− .07	0.45	− 0.15	0.88	− 0.96	0.82
	*X* × *W*	**0.60**	**0.28**	**2.17**	**.03**	**.05**	**1.15**
*X* = ACEs *Y* = Externalization *W* = Intimacy avoidance	Constant	4.64	2.39	1.94	.05	− .09	9.37
	Adverse experiences	4.12	1.64	2.51	.01	0.87	7.36
	Intimacy avoidance	0.42	0.64	0.65	0.52	− 0.85	1.69
	*X* × *W*	− 0.33	0.48	− 0.69	0.49	− 1.27	0.62

Statistically significant interactions are highlighted in bold. *β* regression coefficients, *SE* standard error, *LLCI* low-level confidence intervals, *ULCI* upper-level confidence intervals, *ACEs* adverse childhood experiences

## Discussion

The main objective of the present study was to assess the moderating role of attachment between ACEs and current psychopathology in adolescents from the extreme north of Chile. Generally speaking, of the five hypotheses proposed, two were accepted. The first hypothesis (H1) confirms the relationship between ACEs (parental divorce, mental illnesses in caregivers, exposure to family violence, parental neglect, imprisonment of a household member, and cohabitation with individuals with alcohol- or drug-related issues) and psychopathology; specifically, it was found that as adolescents report a more significant number of ACEs in childhood, the presence of internalizing symptoms (depression/anxiety, social problems, somatic complaints) and externalizing symptoms (aggressive behavior, delinquent behavior, attention seeking) also increases. These findings are similar to studies that have previously linked ACEs with internalizing symptoms such as depression, anxiety, suicidal tendencies, and cognitive impairment (Anderson et al., 2022; Elmore & Crouch, 2020; Karcher et al., 2020; Rytilä-Manninen, 2018) and with externalizing symptoms such as aggressive behaviors and rule-breaking (Anderson et al., 2022). Moreover, these results support other research that has detected a relationship between ACEs and internalizing and externalizing symptoms simultaneously (Bevilacqua et al., 2021). In this context, the relationship between ACEs and psychopathology has been explained from the understanding of ACEs as experiences of high stress or traumatic stress in childhood and how they challenge the psychological resources that children have to manage the reactions (neurophysiological, cognitive, and emotional) generated by these high levels of stress, surpassing and significantly altering their normal functioning.

Furthermore, suppose this stress is chronically present. In that case, it could cause a cumulative risk effect (Evans et al., 2013), affecting their psychological and social adjustment, thus increasing the risk of developing psychopathology (Stein et al., 2022). The allostatic load process would also explain this phenomenon since people continuously exposed to ACEs would see their allostatic load (our body’s capacity to recover from a stressful event) increase, hindering the organism’s homeostasis. This situation would lead to an imbalance of the stress system, which would lead to the manifestation of physical illnesses and mental health problems (Cicchetti, 2011). In this sense, the underlying functioning mechanism would be based on the effect of ACEs in reducing the coping people’s threshold in the face of subsequent severe stressors. Additionally, these experiences would primarily unfold during infancy in a caregiving environment, potentially influencing the formation of attachment styles (Boullier & Blair, 2018; Bowlby, 2003).

Regarding the hypotheses related to the negative impact of ACEs and the moderating role of attachment insecurity, we only accepted hypothesis (H3), which established that the negative impact of ACEs on psychopathology expressed in externalizing symptoms was more significant the more attachment insecurity the adolescents presented, i.e., when they reported more significant abandonment anxiety in their interpersonal relationships. These results contribute to the development of the attachment theory (Bowlby, 2003), particularly to its presence in the adolescent stage, corroborating that insecure attachment is a risk factor for certain psychopathologies in adolescence (Kerns & Brumariu, 2014; Leben Novak et al., 2023). Furthermore, the effect of ACEs did not exert any influence on externalizing symptoms among individuals with low levels of abandonment anxiety. These findings underscore the potential moderating role of abandonment anxiety in the relationship between ACEs and psychopathological outcomes, suggesting that the presence of secure attachment might serve as a protective factor against certain psychopathologies during adolescence (Colonnesi et al., 2011; Groh et al., 2017).

On the other hand, hypotheses (H2) and (H4) were rejected as we did not find sufficient statistical evidence to support a moderating effect of attachment anxiety on the relationship between ACEs and internalizing symptoms, such as depression/anxiety, social issues, and somatic complaints. Similarly, hypothesis (H5) was also rejected under the same statistical criterion, as we failed to observe a moderating effect of intimacy avoidance on the relationship between ACEs and externalizing symptoms, such as aggressive behavior, delinquent behavior, and attention seeking.

It is worth noting that our sample was relatively small, which might have constrained our ability to detect subtler and more intricate moderation effects. Despite hypotheses (H2), (H4), and (H5) not being confirmed based on stringent statistical criteria, the possibility remains that these moderation relationships could be present within the population, albeit not evident within our limited sample size. It is conceivable that, with a larger sample size, we could yield different outcomes and potentially affirm the presence of these moderation effects. Subsequent research endeavors could delve into these relationships using more sizable samples.

The findings of the present study may diverge from previous investigations on ACEs, attachment, and psychopathology in adolescents. Previous investigations have found that attachment insecurity (characterized by high abandonment anxiety and high intimacy avoidance) has a moderating effect on the negative impact of ACEs on internalizing and externalizing symptoms (Corcoran & McNulty, 2018). However, these findings are consistent with studies conducted in adults, wherein a moderating effect of anxious attachment (and not an avoidant attachment) has been observed in the relationship between adverse childhood experiences (trauma) and psychiatric disorders (Bedoya Cardona, 2015; Morán-Kneer et al., 2022). Furthermore, studies such as that of Lacasa et al. (2015) suggest that attachment characterized by preoccupied style (anxiety) is a predictor of externalizing and internalizing symptoms. Additionally, Lin et al. (2020) demonstrated that attachment anxiety can mediate the relationship between ACEs and somatic symptomatology (internalizing symptom).

These differences may be explained by the types of ACEs considered in this study. The present study evaluated only some ACEs, leaving out the ACEs that have been shown to be more invasive and exploitative, such as physical maltreatment, psychological/emotional active maltreatment, and sexual abuse (Emery et al., 2022). This exclusion leads us to reflect on a differential impact between ACEs and psychopathology (especially in externalizing symptoms), considering a specific type of attachment insecurity (abandonment anxiety) as a moderator.

Thus, it could be established that the accumulation of ACEs (parental divorce, mental illnesses in caregivers, exposure to family violence, parental neglect, imprisonment of a household member, and cohabitation with members with alcohol- or drug issues) is related to attachment insecurity of the anxious type and, at the same time, has a negative impact on adolescents, favoring the appearance of externalizing psychopathological symptoms. It should be mentioned that, except for neglect, all these ACEs are indirect, i.e., the child was not the direct object of the adverse or violent experiences, but rather, these experiences occurred to their caregivers and/or family members. In that context, we can hypothesize that if all ACEs were considered, including the more invasive/exploitative ones such as psychological/physical maltreatment and sexual abuse, the attachment disorganization could have been higher (more anxiety and more avoidance at the same time) exerting a more impact on psychopathology (greater internalizing and externalizing symptomatology in adolescents (Granqvist et al., 2017).

Future research lines should confirm whether, indeed, using a broad spectrum of adverse experiences, including more invasive experiences (such as sexual abuse and child maltreatment), may have an even more negative effect on attachment and psychopathology, i.e., increased attachment disorganization and also diverse effects on both internalizing and externalizing symptomatology as well as psychopathological manifestations.

Three main limitations of the study will be pointed out. Firstly, the data collected came from self-report tests, which could have the effects of acquiescence bias and social desirability (Morales Vallejo, 2000). Secondly, the study was conducted under a cross-sectional design in data collection, with the inherent limitations of this type of study; thus, future longitudinal studies could provide more robust information on this topic by following up on ACEs and psychopathology in adolescents. Thirdly, the sample used pertains to a specific region of Chile (North), limiting the generalization of results to other groups of Chilean adolescents and different contexts. Consequently, the results should be viewed with caution. Addressing these limitations through future research would undoubtedly contribute to a more comprehensive understanding of the complexities surrounding ACEs and their potential impact on psychopathology in adolescents.

### Practical implications

The present study’s findings may have significant practical implications for public policy in adolescents’ mental health, specifically for the “Integral Health for Adolescents and Youngs Program” of the Chilean Health Ministry. It is crucial for professionals working in the clinical and psychosocial area to consider the effect ACEs may have on child and adolescent mental health. The aim would not be to modify them but rather to develop a comprehensive view of psychopathology and child and adolescent mental health that goes beyond uni-causal explanations, gathering the life experiences of adolescents and how they narrate or rewrite their biography and the influence they attribute to these adverse experiences and their consequences, integrating them into their life histories. Similarly, the accumulation of  early caregiving experiences and an examination of their significance for adolescents can help us understand how these experiences may influence protective or risk factors in the development of psychopathology. Additionally, the collected information could be used to support programs based on attachment theory, which aims to repair and re-signify childhood attachment experiences with the new bonds they establish from this stage of the life cycle onwards, including the bond with caregivers. 

## Conclusion

The results of the present study indicate that the accumulation of ACEs (parental divorce, mental illnesses in caregivers, exposure to family violence, parental neglect, imprisonment of a household member, and cohabitation with individuals with alcohol- or drug-related issues) is associated with the development of psychopathology in adolescents from northern Chile, specifically, with the presence of internalizing and externalizing symptoms. Regarding moderation analyses, attachment insecurity, as represented by abandonment anxiety, moderates the relationship between ACEs and externalizing symptoms. These findings suggest that lower levels of abandonment anxiety could mitigate the effects of ACEs on adolescent psychopathology, while higher levels of abandonment anxiety could enhance these effects on adolescent psychopathology. These results could be considered in the formulation of clinical interventions in mental health programs aimed at adolescents. Such interventions should address both risk and protective factors, including childhood experiences and caregiving relationships to which adolescents have been exposed.

## Data Availability

All data related to this study are available from the authors upon request.
